# Fatigue database of complex metallic alloys

**DOI:** 10.1038/s41597-023-02354-1

**Published:** 2023-07-12

**Authors:** Zian Zhang, Haoxuan Tang, Zhiping Xu

**Affiliations:** Applied Mechanics Laboratory and Department of Engineering Mechanics, Tsinghua University, Beijing, 100084 China

**Keywords:** Metals and alloys, Industry

## Abstract

The past few decades have witnessed rapid progresses in the research and development of complex metallic alloys such as metallic glasses and multi-principal element alloys, which offer new solutions to tackle engineering problems of materials such as the strength-toughness conflict and deployment in harsh environments and/or for long-term service. A fatigue database (FatigueData-CMA2022) is compiled from the literature by the end of 2022. Data for both metallic glasses and multi-principal element alloys are included and analyzed for their statistics and patterns. Automatic extraction and manual examination are combined in the workflow to improve the efficiency of processing, the quality of published data, and the reusability. The database contains 272 fatigue datasets of *S*-*N* (the stress-life relation), *ε*-*N* (the strain-life relation), and d*a*/d*N*-Δ*K* (the relation between the fatigue crack growth rate and the stress intensity factor range) data, together with the information of materials, processing and testing conditions, and mechanical properties. The database and scripts are released in open repositories, which are designed in formats that can be continuously expanded and updated.

## Background & Summary

**Fig. 1:**
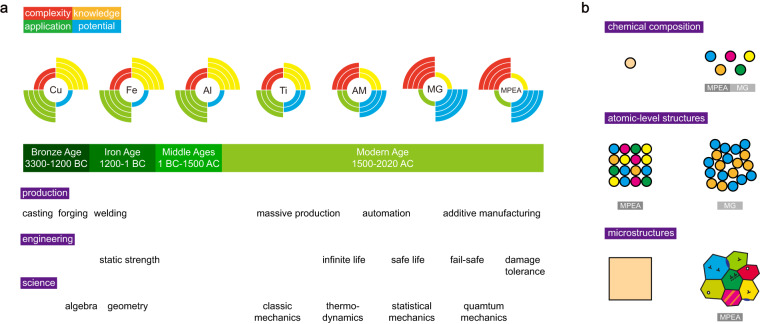
Complexity of metallic alloys. (**a**) The development of metallic alloys, production technology, engineering design criteria, and science. The timeline is scaled for different periods for the clarity of presentation. Each circular barplot presents the levels of four properties of the metallic alloys with its name marked in the center. ‘AM’ denotes the additively manufactured alloys, ‘MG’ denotes the metallic glass, and ‘MPEA’ denotes the multi-principal element alloy. The upper-left quarter denotes the complexity of the material, as illustrated in panel b. The upper-right quarter denotes the human knowledge of the material. The lower-left quarter denotes the range of applications of the material. The lower-right quarter denotes the potential of the material to be developed with superior mechanical performance. (**b**) The complexity of metallic alloys includes chemical composition, atomic-level structures, and microstructures. The complexity of MGs and MPEAs in each aspect is highlighted.

Metallic materials are so important that the historical development of human civilization can be represented by their usage (Fig. 1a). Research and development of metallic alloys embody and promote the advances in material sciences, experimental tools, and manufacturing processes. Early development of advanced alloys is largely empirically, by trial and error. Theoretical and numerical methods^1–4^ based on the physics and chemistry of metals were gradually established in the 20th century. However, it remains challenging to resolve the complexity that relates the microstructures of metallic alloys to their performance. Even in the limit of single crystals (SC) where the grain boundaries (GBs) are eliminated for the gain of exceptional high-temperature mechanical performance^5^, defects such as the dislocation networks evolve with plastic deformation and disturb the perfection of crystalline structures. Recently, the exploration of materials with predesigned nano- or microstructures such as nano-crystalline (NC), nanotwinned (NT), and functionally-graded (FG) alloys finds great success in discovering high-performance alloys. For example, GB strengthening (also known as the Hall-Petch effect^6,7^) guides the development of high-strength alloys by refining the grains to a specific level^8^. Research has also been devoted to the development of complex metallic alloys such as metallic glasses (MGs) and those with multiple principal elements are proposed and manufactured^9,10^. The chemical and structural heterogeneity is shown to be able to hinder crystallographic slips and improve the strength and fracture toughness^11,12^.

The complexity of metallic alloys stems from many aspects (Fig. 1b). The chemical composition of principal elements and their atomic-level structures control the electronic structures and orders in different ranges (short, medium, or long)^13,14^. The nature of chemical bonding and lattice symmetries are then defined, which play dominant roles in the mechanical behaviors of materials. Specifically, atomic-level structures such as body-centered cubic (BCC), face-centered cubic (FCC), hexagonal close-packed (HCP), and amorphous structures define the local arrangement of atoms and long-range orders of translation and rotation. These features are crucial for the slip processes that govern the plasticity of materials. For example, slip localization is more prominent in FCC and HCP than BCC alloys, resulting in lower normalized fatigue strength (FS/YS or FS/UTS) in the former class^15^, where FS, YS, and UTS are abbreviations of fatigue strength, yield strength, and ultimate tensile strength respectively. Furthermore, alloying elements can strengthen the materials by forming solid solutions or precipitation^16^ and even inducing phase transformation^17^. The plasticity in amorphous structures proceeds via shear transformation or diffusive-jump processes^18^, which are associated with local atomic rearrangement. The activation energy of local atomic rearrangement is usually higher than that of crystalline slips and thus results in elevated strength in MGs^18,19^. The ‘material genes’ such as the composition, atomic-level structures, and microstructures unfold a hyper-dimensional space of design for the discovery of complex metallic alloys as well as a wide spectrum of promising performance. By following a data-driven approach, such exploration can be much expedited.

The design of alloy composition is limited by the thermodynamic stabilities of alloying phases with specific atomic-level structures^1^. Microstructures such as the texture depend largely on the synthesis procedures and thermomechanical processing conditions. For example, FCC austenite, BCC ferrites, and body-centered tetragonal (BCT) martensites are three major phases of steels, as controlled by the FCC-preferred Ni equivalent and BCC-preferred Cr equivalent of alloying elements^20^. HCP α-Ti can be stabilized by Al, O, N, and C whereas BCC β-Ti can be stabilized by V, Mo, Ta, and Nb^21^. The composition also influences the phase fraction in dual-phase (DP or duplex) alloys such as DP steels and α + β titanium alloys^21,22^. In alloy systems such as TiAl and NiAl, intermetallics with different lattice structures form as the composition varies, allowing the achievement of high strength^21,23^. For Ni-based SC superalloys, intermetallics γ′ phases of Ni_3_ (Al, Ta, Ti) in the L1_2_ structures are dispersed in a γ matrix (FCC Ni), offering extraordinary high-temperature strength^5^. The size, morphology, and texture of grains can be controlled by the thermomechanical processing conditions (temperatures, loads, and their sequences)^24,25^. Recently, additive manufacturing (AM) has been widely explored for its facile fabrication of structural components with complex geometries. The control of microstructures in AM can be established in specific regions at the micrometer scale under laser or electron beams^26–28^ and characterized *in situ*^29,30^. In addition, advanced techniques such as directional solidification (DS) and severe plastic deformation (SPD) processing are used to prepare SC and NC alloys, respectively^31,32^. Conventional fabrication techniques for alloys with low or medium complexities (e.g., single- or few principal elements, polycrystalline textures) are widely deployed because of their high technology readiness levels (TRLs) and low costs. To further elevate the alloy performance and lift the strength-toughness conflict, key insights into the composition-processing-microstructures-performance (CPMP) relationship are needed, especially for alloys with high complexities in the chemical composition and/or atomic-level structures.

MGs and multi-principal element alloys (MPEAs) are two emerging metallic materials featuring high compositional and structural complexity. The discovery of metallic glasses in 1960 overturns our knowledge of metals, which was largely limited to crystalline materials at the time^9^. The development of alloys with deep eutectics since the 1990s further allows liquid-like structures to be retained in thicker sections in the amorphous state on cooling to ambient temperature^33^. The amorphous structures of MGs represent the extreme of the complex atomic-level structures, offering high strength and sometimes high fracture toughness^12^. On the other hand, the studies of multi-principal element alloys (MPEAs) date back to the 1970s and similar concepts appeared subsequently, such as high-entropy alloys (HEAs), and complex concentrated alloys (CCAs)^34^. MPEAs explored in this work cover metals with multiple (≥3) principal elements in a single phase of solid solution, in contrast to precipitations in conventional alloys. MPEAs are compositionally complex but with relatively simple atomic-level structures. The interatomic interaction is, in general, a mixture of metallic, ionic, and covalent nature. MPEAs usually feature excellent thermal stability and high-temperature strength due to the strong bonding between the atoms and the low diffusivity of atoms for the high degree of long-range orders^35^. MGs and MPEAs demonstrate different aspects of material complexities and form representative subsets of advanced alloys, the research of which is still in the stage of exploration. Establishing well-structured databases for complex MGs and MPEAs and keeping them up to date by subsequent development are thus crucial for the data-driven approach^36^, which can facilitate exploration of the composition space and optimization of the processing conditions, towards improvement in the alloy performance^37^.

Data science is of crucial importance in the research of MGs and MPEAs for the vast design space compared to conventional alloys. Experimentally measured^36,38,39^ and theoretically predicted^40,41^ data reported in the literature and research reports are valuable sources to understand and improve the performance of alloys. Text mining, image data processing, and artificial intelligence^37,42^ techniques are expected to offer insights into the CPMP relationship as demonstrated in our recent work on AM alloys^43^. However, constructing such a large database involves processes of data generation, collection, publication, and maintenance, which are time-, cost-, and labor-intensive and beyond the capability of a single researcher or group. Worldwide cooperation under open science and FAIR (findable, accessible, interoperable, and reusable) principles becomes a promising solution for this issue^44^. It should be noted that there are several intrinsic characteristics of data in materials sciences. For example, material properties of crystals and polymers can be predicted through high-throughput theoretical calculations, even at the first-principles level^45,46^. Experimental data for these materials, in addition to the role of the data themselves, can also be used for the verification and validation (V&V) of theoretical models. However, material properties such as fatigue cannot be predicted a prior accurately by theory due to the complex paths of microstructural evolution^47,48^, and experimental data become the only reliable source.

Experimental studies report extraordinary mechanical properties for MGs and MPEAs^18,49,50^, which show advantages in harsh service conditions such as fatigue and corrosion^11,49,51^, where conventional alloys already reach their limits. For example, components made from MGs and MPEAs are promising for space exploration and settlement^52^, which require materials to withstand extreme conditions of heat, impact, and radiation during their full phases of service^53^. However, applications of MGs and MPEAs in the design of structural integrity are still preliminary due to the limited size of MGs, cost of production, and incompatibility with conventional industrial manufacturing processes. One of the crucial performances of advanced alloys is the resistance to long-time and mostly variable, or namely fatigue, loading conditions. Fatigue has been studied for more than one century^54^ but still plagues scientists and engineers, and causes substantial loss^55,56^. In contrast to the stiffness, strength, and toughness, fatigue performance measured from experiments of alloys usually display large dispersion. To ensure the reliability in industrial applications, standards^57–60^ and guidelines^61^ are developed for the design, tests, fabrication, operations, and maintenance of fatigue-susceptive components. These regulations provide a baseline for the evaluation of fatigue properties using data of the stress-life (*S*-*N*), strain-life (*ε*-*N*), and fatigue crack growth rates (FCGR, d*a*/d*N*-Δ*K*) relations for high-cycle fatigue (HCF), low-cycle fatigue (LCF) and damage tolerance design, respectively. It is thus necessary to construct a fatigue database for complex metallic alloys such as MGs and MPEAs.

In this work, we collect fatigue data and related information reported for MGs and MPEAs in 1,249 scientific articles (up to the end of 2022). Open-source and in-house codes are used for data extraction from figures, tables, and text. The description of research and reported *S*-*N*, *ε*-*N* and d*a*/d*N*-Δ*K* data are outlined.

## Methods

Our workflow includes content acquisition (search and download), data extraction (from figures, tables, and text), and database construction. A brief description of the workflow is given below. Only the key points and improvements of our methodology are reported here, and details can be found in our previous work on the fatigue data of AM alloys^43^. The performance of automated data extraction is assessed by the metrics, precision, recall, and F1 score, that are

1 $${\rm{precision}}=\frac{{\rm{TP}}}{{\rm{TP}}+{\rm{FP}}},$$

2 $${\rm{recall}}=\frac{{\rm{TP}}}{{\rm{TP}}+{\rm{FN}}},$$

3 $${\rm{F}}1=2\frac{{\rm{precision}}\times {\rm{recall}}}{{\rm{precision}}+{\rm{recall}}},$$

where TP denotes the true positive or the number of correctly-extracted data, FP is the false positive or the number of incorrectly-extracted data, and FN is the false negative or the number of data that are not extracted. The F1 score is the harmonic mean of precision and recall.

### Content acquisition

Keywords for MGs, MPEAs, and fatigue are summarized (Table 1) and compiled into the search formulas. WoS returns 1,249 records of articles. Irrelevant articles are filtered out by a natural language processing (NLP) classification model^62^. The records are then corrected by manual examination. 326 targeted articles are downloaded according to their digital object identifiers (DOIs) by using publisher application programming interfaces (APIs) and the open-source code, article-downloader^63^. Documents in formats of the extensible markup language (XML)/hypertext markup language (HTML) and portable document format (PDF) are both downloaded, which are then processed by automated parsing and manual examination, respectively.

**Table 1:** Keywords used for article search in the citation database.

Category	Keyword
Fatigue	fatigue
Metallic glass	metallic glass/BMG/amorphous metal/amorphous alloy/non-crystal metal/non-crystal alloy/disorder metal/disorder alloy
Multi-principal element alloy	multi-principal element alloy/MPEA/high-entropy alloy/HEA/medium-entropy alloy/MEA/multicomponent alloy/multi-element alloy/complex concentrated alloy/CCA/baseless alloy

### Data extraction

**Fig. 2:**
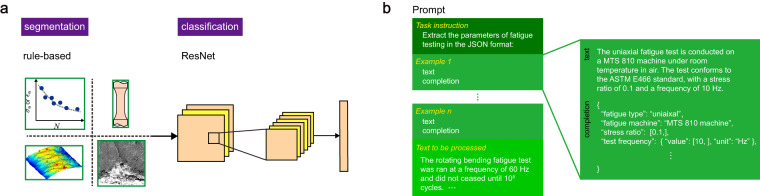
Figure processing and text data extraction. (**a**) Figures from articles are first segmented by a rule-based script and then classified by using a convolutional neural network (CNN) model, ResNet^64^. (**b**) A prompt is designed for GPT-3.5^67^ with the task instruction, examples, and text to be processed. To evaluate the performance of few-shot learning, 2-3 examples are used.

Figures are extracted from the PDF documents using PyMuPDF. Segmentation of figures with multiple panels is automated by a rule-based code, reaching an F1 score of 86% (Table 2). Classification of fatigue figures is conducted by a convolutional neural network (CNN) model, ResNet^64^ (Fig. 2a), with an F1 score of 90% (Table 2). The fatigue data (*S*-*N*, *ε*-*N*, and d*a*/d*N*-Δ*K*) presented in scatter plots are extracted by an in-house MATLAB code IMageEXtractor (IMEX)^43^. Tables in XML/HTML files are parsed by table extractor^65^ whereas those embedded in the PDFs are processed manually. The evaluation metrics of table data extraction are summarized in Table 2.

**Table 2:** Evaluation metrics of automated data processing.

Source	Function	Precision	Recall	F1
figure	figure segmentation	81%	90%	86%
	figure classification	86%	94%	90%
	data extraction	82%	51%	63%
table	data extraction	62%	76%	68%
text	data extraction	81%	97%	88%

Texts in the XML/HTML format are extracted using our in-house parsing codes TEXTract^43^. Articles with only PDF files available are extracted by the PDFDataExtractor^66^. Text classification is conducted for abstracts and paragraphs using the NLP library Simple Transformers with Robustly Optimized BERT Pretraining Approach (RoBERTa)^62^. Data including the information on materials, processing, testing, and mechanical properties are extracted from target paragraphs by employing the Generative Pre-trained Transformer (GPT)^67^ of version 3.5. GPT is a large pre-trained language model with billions of parameters and excellent performance of few-shot learning^67^. GPT can conduct a text completion task that generates the most probable completion (output) based on the prompt from users. Our prompts in GPT include a task instruction, examples, and the text to be processed (Fig. 2b). The task instruction asks the GPT to extract data from text and return them in JSON format. Each example is a text-completion pair to demonstrate the content and format of the data to be extracted. The text to be processed is placed at the end of the prompt. GPT will then return the completion formatted in JSON. Since the maximum number of tokens is 4,096 (including the prompt and completion) in the current release, the work here is conducted by processing the paragraphs one by one provided with 2-3 examples (few-shot learning). The F1 score of GPT-3.5 is 88%, suggesting that large language models (LLMs) such as GPT are able to understand most of the text and reduce the human work on constructing extraction rules or building NLP models. Fine-tuned or upgraded GPT models may improve the F1 score but the performance may still be insufficient to construct a high-quality, research-level database from the literature^68–70^. Besides, GPT of versions >3 is not open-sourced. We thus do not fine-tune the GPT model and proceed with manual data correction. The product data can be used as training sets for GPT and alternative LLMs such as LLaLMA^71^ and GLM^72^.

### Database integration and data correction

To construct the database, fatigue data extracted from figures should be correlated with data entries of material information, processing and testing conditions, and mechanical properties extracted from text and tables. Most of the data entries such as processing and testing parameters do not vary in the same article, and single values extracted for a specific data entry are assigned to all datasets related to the article. For data entries with multiple values, the assignment is made according to the legend labels.

Fatigue data of critical structural components in service are sensitive to processing, loads, and environment. Manual examination and correction are thus necessary to ensure the quality of data. The amount of manual work depends on the performance of the automated workflow and the requirement for the accuracy of specific applications. For design purposes, data should be accurate for reliable components and manual corrections are essential. Although the quality of data released with this paper is ensured by manual corrections, we note that for the use of the database in machine-learning models, a limited number of false values can be tolerated. Comprehensive inspection can thus be replaced by sampling inspection, which significantly saves labor work considering the large volume of the scientific literature corpus. To guide the construction of a high-quality database, the sampling strategy is determined based on the accuracy of automated data extraction (*α*_a_) or manual examination (*α*_m_) reported in our work and other high-quality literature-informed database^36,38,39,43^. For a target accuracy (*α*_t_), the size of sampling set relative to the complete datasets (*s*) and the rounds of examination (*n*_r_) are determined by the condition of $\left(1-{\alpha }_{{\rm{a}}}\right){\left[1-s+s\left(1-{\alpha }_{{\rm{m}}}\right)\right]}^{{n}_{{\rm{r}}}}\le 1-{\alpha }_{{\rm{t}}}$.

## Data Records

**Fig. 3:**
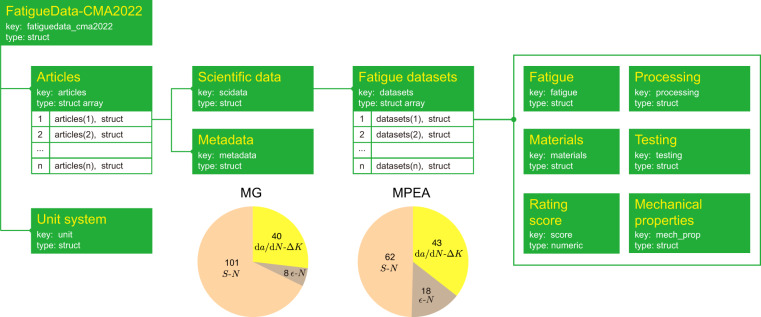
Database structure. The FatigueData-CMA2022 database is formatted into a hierarchical tree structure. The name of each tree node is highlighted in yellow color. Keys are defined for easy access by scripts. Each node has its specific data type. Two pie charts show the statistics of the types of fatigue datasets for metallic glasses (MGs) and multi-principal element alloys (MPEAs).

The FatigueData-CMA2022 database^73^ is available as MAT (MATLAB), JSON, and EXCEL files at 10.6084/m9.figshare.23007362. MGs and MPEAs applied as coatings for other substrate materials are excluded since the data do not represent their performance^74^. The MAT and JSON files are formatted into a hierarchical tree structure, as shown in Fig. 3. The tree nodes that store data values are called data entries. Data entries include string and numeric data types. Text data such as titles, types of processing, and fatigue testing are stored as strings. Data with multiple strings such as authors, countries, and institutions are stored as string arrays. The year of publication is defined as a numeric number, and other numeric data such as fatigue data, parameters of processing, and load ratios are stored in the form of numeric arrays. The tree nodes used to group data entries are called data structs. Multiple structs such as articles or fatigue datasets are arranged into struct arrays. To facilitate programming implementation and data acquisition, keys are defined for data entries, structs, and struct arrays (Fig. 3, Tables 3–5).

**Table 3:** Contents of the struct of ‘metadata’.

Struct	Data Entry	Data Key	Data Type
Metadata			
	Title	title	string
	Authors	author	string array
	Source of the publication	source	string
	Year of publication	year	numeric
	Institution	institution	string array
	Country and region	country_region	string array
	Funding agency	fund	string array
	DOI	doi	string

**Table 4:** Contents of the struct in the ‘fatigue datasets’ struct array.

Struct	Data Entry/Struct	Data Key	Data Type
Fatigue			
	Fatigue data	fat_data	numeric
	Types of fatigue data	fdata_type	string
	Method of extraction	extract_method	string
Materials			
	Type of the material	mat_type	string
	Original name of the material	mat_name	string
	Name of the material sorted by composition	mat_name2	string
	Type of the element ratio of ‘mat_name’	ratio_type	string
	Atomic-level structure	atomic_struct	string
	Composition	composition	numeric
	Glass transition temperature	glass_tran	numeric
	Size of grains	grain_size	numeric
Processing			
	Processing parameters	proc_para	struct array
	Processing sequence	proc_seq	numeric
	Surface treatment parameters	surf_para	struct array
	Surface treatment sequence	surf_seq	numeric
	Ingot description	ingot_desc	string
	Size of ingot	ingot_size	numeric
Testing			
	Types of fatigue tests	fat_type	string
	Fatigue temperature	fat_temp	numeric
	Fatigue environment	fat_env	string
	Load ratio	fat_r	numeric
	Frequency	frequency	numeric
	Fatigue machine	fat_machine	string
	Fatigue standard	fat_standard	string
	Specimens description	spec_desc	string
	Critical cross-section size of specimens	spec_size	numeric
	Stress concentration factor of specimens	spec_kt	numeric
	Load control	load_ctrl	string
	Failure criterion	fail_crt	string
Mechanical properties			
	Young’s modulus	modulus	numeric
	Yield strength	yield_strength	numeric
	Ultimate tensile strength	ultimate_strength	numeric
	Elongation	elongation	numeric
	Fracture toughness	toughness	numeric
	Fatigue crack growth threshold	kth	numeric

**Table 5:** Contents of the struct in the ‘processing parameters’ and ‘surface treatment parameters’ struct arrays, dependent on the types of processing.

Types of processing	Data Entry	Data Key	Data Type
For all			
	Type	type	string
Heat treatment			
	Temperature	temperature	numeric
	Time	time	numeric
Remelting			
	Times of remelt	times	string
Casting			
	Type of mold	mold	string
Rolling/forging/swaging			
	Initial size	from	numeric
	Final size	to	numeric
	Reduction of diameter	diameter_reduction	numeric
	Reduction of thickness	thick_reduction	numeric
Surface treatment			
	Method	method	string

The top-level data structures of the FatigueData-CMA2022 database^73^ are shown in Fig. 3. The root node is the database, containing children nodes of articles and a default unit system (e.g. MPa for stress, °C for temperature). Raw numeric data are converted to default units of data entries. Articles are stored in a struct array. Each article contains two structs of metadata and scientific data. Metadata contains data entries such as the titles and authors of articles. Scientific data store a struct array of fatigue datasets. Each dataset is obtained from experimental tests under different conditions. A fatigue dataset contains 5 structs (fatigue, materials, processing, testing, and mechanical properties) defined in Table 4 and a rating score is defined to measure the quality of data. The processing parameters are organized as a struct array, ‘proc_para’, and the content of each struct depends on the types of material processing. The processing sequence is recorded in the ‘proc_seq’ array with the index in ‘proc_para’. For surface treatments, ‘surf_para’ and ‘surf_seq’ are defined in the same way as processing parameters and sequence. More information on the specimen surfaces such as surface roughness, residual stress, and surface defects, are not included in the current database since not many of the published data include the information, which shows the need for standards of fatigue performance reporting. However, the information can be added to the database in the future through the API.

The terminology of data types is largely inherited from MATLAB (the MAT file). Exceptions are string arrays and struct arrays of processing parameters and surface treatments, which correspond to cell arrays in the MAT file. For the JSON file, the struct is defined as a dictionary, and all types of arrays are defined as lists. The database is also flattened into an EXCEL file, including 4 worksheets. The worksheets of ‘S-N’, ‘e-N’, and ‘dadn’ store *S*-*N*, *ε*-*N*, and d*a*/d*N*-Δ*K* data, respectively. In these 3 worksheets, each row stores the index of a fatigue dataset and a data descriptor (*S*/*ε*, *N*, and the run-out flag for ‘S-N’/‘e-N’, d*a*/d*N* and Δ*K* for ‘dadn’). The d*a*/d*N*-Δ*K* data extracted by colors store all matched pixels. The number of data points exceeds the maximum number of rows allowed by EXCEL (1,048,576). As a result, 500 data points are uniformly sampled from each dataset and then recorded. In the 4th worksheet of ‘parameter’, each row stores the index of a fatigue dataset and its contents. Each column corresponds to a data entry. Data in the ‘parameter’ worksheet is linked to the other three through the index of fatigue datasets.

With the structures of the datasets outlined above, the data entries are explained here in more detail. The ‘fatigue data’ array store *N* or Δ*K* in the first column, and the values of *σ*_a_, *ε*_a_ or d*a*/d*N* in the second column. *ε*_a_ stands for the amplitude of total strain including the elastic or plastic components. The third column stores the run-out flag for *S*-*N* and *ε*-*N* data, where ‘1’ denotes that the test stops before failure (run-out) and ‘0’ denotes failure. The size of the critical cross-section stores the diameter for specimens with circular cross-sections, the outer and inner diameters for those with annular cross-sections, and the width and thickness for those rectangular cross-sections, respectively. The size of ingots is defined in the same way, with the longitudinal size added. The shapes are stored in the description of specimens (‘spec_desc’) and ingot (‘ingot_desc’). The stress concentration factors (*K*_t_) of specimens are recorded, and the stress in ‘fatigue data’ is the nominal stress without multiplying the *K*_t_. In the numeric arrays of other data entries, a single value stands for a specific value or the mean, and two values stand for the lower and upper bounds, respectively. For the convenience of comparison between the string data, unified nomenclature is used for data entries such as types of processing, materials, machines, affiliations, and funding agencies.

In our database, data entries not reported explicitly are recorded as empty arrays (MAT), lists (JSON), strings (MAT and JSON), or cells (EXCEL). We assume that the testing is uniaxial and conducted under an ambient environment (25 °C, air) with *K*_t_ = 1 if not specified. The default load control is ‘force’ for *S*-*N*, ‘strain’ for *ε*-*N*, ‘load’ for d*a*/d*N*-Δ*K*. The total fatigue life is defined according to failure criteria, such as the fracture of specimens and the percent of load drop. Fracture of specimens is assumed as the default value, as added into the database as ‘fracture’.

**Fig. 4:**
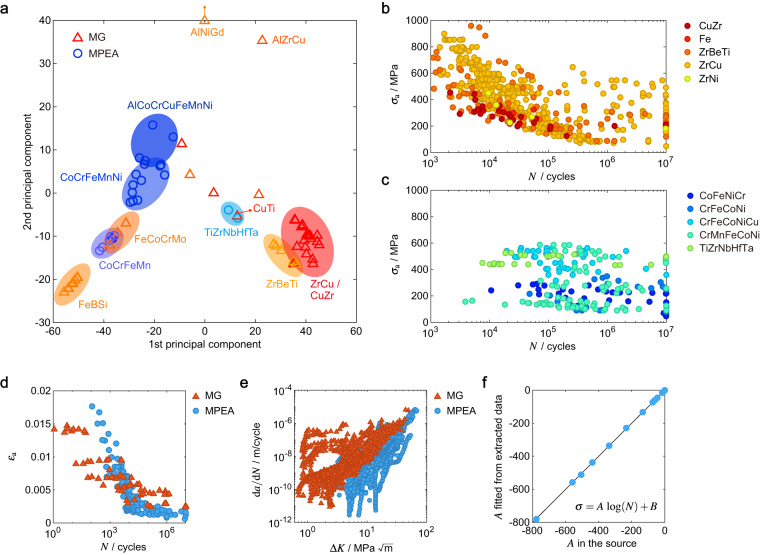
Representative Data. (**a**) Chemical composition of metallic glasses (MGs) and multi-principal element alloys (MPEAs) are presented in the space spanned by their 1st and 2nd principal components. Note that the chemical formula in the annotation is the union of principal elements for the alloys grouped by ellipses. Representative *S*-*N* data of (**b**) MGs and (**c**) MPEAs at the stress ratio of *R* = 0.1. Representative (**d**) *ε*-*N* and (**e**) d*a*/d*N*-Δ*K* data of MGs and MPEAs for all *R*. (**f**) Comparison between fitting parameters of the extracted *S*-*N* data and those in the source publications.

In summary, the database covers 51 types of MGs and 25 types of MPEAs. For MGs, 47 articles report 101 *S*-*N* datasets with 989 data points, 2 articles report 8 *ε*-*N* datasets with 63 data points, and 18 articles report 40 d*a*/d*N*-Δ*K* datasets (Fig. 4). For MPEAs, 36 articles report 62 *S*-*N* datasets with 503 data points, 10 articles report 18 *ε*-*N* datasets with 174 data points, and 14 articles report 43 d*a*/d*N*-Δ*K* datasets (Fig. 4).

## Technical Validation

The performance metrics of figure, table, and text processing show that the F1 scores of automated extraction are 60–90% (Table 2). Although automated extraction methods can be further improved, the production of high-quality databases is still challenging. Considering that a fatigue database is critical and urgent for the development of advanced alloys MGs and MPEAs, all data records are manually examined and corrected to ensure the quality of the database in this work. Subsequent inspection of 50 randomly chosen articles shows that the precision is improved to be >98%. We include 61/65 datasets of the recent collection of HEA fatigue data^38^. There are 4 datasets not included for the data are not reported in standard *S*-*N* format or the articles are not in the WoS records. These two databases can be used for mutual verification of the common records, which may increase the credibility of open data for the end users. We find that both the contents and formats of literature-derived databases can be different and complementary due to the different perspectives, demands, and experiences of researchers. Guidelines and standards can help the fusion of these databases. In comparison, more parameters in the metadata are included in our database (e.g. affiliations, countries, funding sources, DOIs), surface conditions, and processing techniques (e.g. thickness reduction of rolling, types of cooling, times of remelting), which are released in more flexible and organized hierarchical formats (JSON and MAT) in addition to the accompanying EXCEL document. The data records of grain size are added to our database as inspired by ref. ^38^.

The database can be examined by domain knowledge. The richness and organization of our database allow us to explore the CPMP relationship. The chemical composition of each unique material is arranged into a vector. All composition vectors are analyzed by principal component analysis (PCA)^75^ and visualized according to the 1st and 2nd principal components in Fig. 4a. The outliers of chemical composition are checked to avoid errors in data extraction. Alloys are then grouped according to their major elements with composition >10%. Figure 4a shows that MGs, and MPEAs form clusters in the space of composition. The major elements of a specific MPEA usually accommodate the same atomic-level structures, such as Co, Cr, Fe, Mn, and Ni for FCC, or Ti, Zr, Nb, Hf, and Ta for BCC. Adding elements with distinctly different atomic-level structures can lead to precipitation^76^. On the other hand, MGs may contain metallic and semiconductor elements with different atomic-level structures from their own crystal phases, which promotes the formation of amorphous structures. MPEAs reported in the literature span over a narrower composition space than MGs, which could be attributed to the difficulties in obtaining single phases of solid solutions with multiple principal elements and the relatively shorter research and development history of MPEAs. The numbers of major elements are generally *n* ≤ 3 for MGs and ≥3 for MPEAs. High-entropy MGs (*n* > 3) are also studied and some of them feature similar chemical composition as those of MPEAs^77^.

Examination of material-specific properties (e.g. fatigue performance, glass transition temperature *T*_g_, mechanical properties) is then conducted for the groups of alloys. Fatigue data are plotted to examine their correlations, which are negative for the *S*-*N* and *ε*-*N* data, and positive for the d*a*/d*N*-Δ*K* data (Fig. 4b–e). Outliers of fatigue data due to errors in data processing are examined visually for each group of materials, and then corrected in IMEX. Representative *S*-*N* data of MGs are shown in Fig. 4b,which are tested under a stress ratio (*R*) of 0.1. The tension-tension loading *R* = 0.1 is used for 68% of the MGs data to avoid buckling of MG specimens featuring small diameters and large aspect ratios, which are commonly used in these studies. *R* = 0.1 is also preferred in 3 or 4-point bending fatigue tests. ZrCu-based MGs are dominant in the database for their good glass-forming abilities (GFA), which are commercially available as products of Vitreloy and LiquidMetal^78^. For CuZr-based MGs where the composition of Cu is higher than Zr, the fatigue performance is lower. This dependence on the chemical composition may be attributed to the difference in the atomic packing density^79^. Representative *S*-*N* data for MPEAs measured at *R* = 0.1 are also summarized for comparison (Fig. 4c). Most data are reported for FCC MPEAs with major elements of Co, Fe, Ni, Cr, and Mn. The fatigue performance of BCC MPEAs (TiZrNbHfTa) is higher than that of FCC MPEAs, which conforms to the understanding of conventional alloys as a result of the atomic-level structures^15,80,81^. The FS of MGs and MPEAs measured in the HCF condition (10^4^–10^7^ cycles) are distributed in a similar range between 100 and 600 MPa. The MPEAs show higher LCF (<10^4^ cycles) performance and better ductility than the MGs (Fig. 4d). The FCGRs of MGs are in general higher than those of MPEAs, showing the difference in brittleness (Fig. 4e). It should be remarked for data processing that, the d*a*/d*N*-Δ*K* data could include the near-threshold regime, the Paris regime, and the unstable growth regime (Fig. 4e). The threshold for fatigue crack growth and fracture toughness are included in the database as a reference for the fatigue crack growth behaviors. For further validation, the fitting parameters obtained from the extracted data are compared to those in the source publications, which show good consistency (Fig. 4f). The negligible errors of fitting parameters may be induced by the distortion of data symbols in the pixelized figures. For the relatively low TRL of fabrication techniques for MGs and MPEAs, imperfections such as defects may exist in the materials under investigation, which can result in strong dispersion of the reported data. The deviation from fatigue data fitting using existing life models can help the users to identify the datasets with large dispersion and the detailed information can be found by referring to the source publications.

**Fig. 5:**
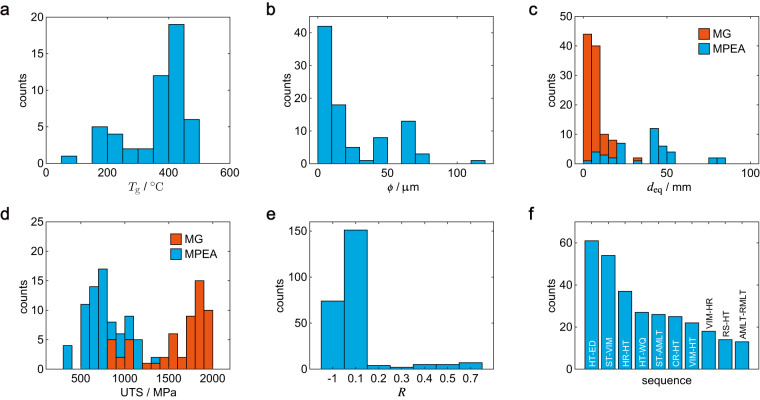
Statistics of material parameters. The histogram of (**a**) glass transition temperatures (*T*_g_) for MGs, (**b**) grain sizes (*ϕ*) for MPEAs, (**c**) equivalent diameters (*d*_eq_) of ingots, (**d**) ultimate tensile strengths (UTS) and (**e**) stress ratios (*R*) for both MGs and MPEAs. (**f**) The top 10 paired steps of the thermomechanical processing sequences for MPEAs. The abbreviations are ST: the start of processing, VIM: vacuum induction melting, AMLT: arc-melting, RMLT: re-melting, HR: hot rolling, CR: cold rolling, RS: rotary swaging, HT: heat treatment, WQ: water quenching and ED: the end of processing.

In addition to the chemical composition and fatigue performance, other data including information on materials, processing and testing conditions, and mechanical properties are examined, which are also valuable to understand the CPMP relationship. Material-specific properties are examined material by material, such as the glass transition temperature (*T*_g_), grain size (*ϕ*), ingot size, and mechanical properties. *T*_g_ ranges from 91 to 480 °C (Fig. 5a), which shows a strong correlation with the type of materials. For example, the value of *T*_g_ is ~90 °C for Ca-based MGs^82^, ~200 °C for Al-based MGs^83^, and ~400 °C for Zr-based MGs^84^. The grain size of HEAs is mainly in the range between 1 and 100 μm (Fig. 5b), which shows stronger dependence on the processing conditions than the types of materials. Samples with sub-micron grains generally underwent special processes such as equal channel angular pressing (ECAP) or friction stir processing (FSP), whereas those with grains larger 100 μm are usually results of high-temperature heat treatment. In addition to microstructures, the size of components made from these materials is also important in applications. The equivalent diameter of ingots is calculated from the area of cross-section, ${d}_{{\rm{e}}{\rm{q}}}=2\sqrt{{\rm{a}}{\rm{r}}{\rm{e}}{\rm{a}}/\pi }$. As shown in Fig. 5c, large ingots can be produced for MPEAs but the high-cooling rate restricts the size of MGs. Lowering the critical cooling rate to produce bulk MGs (BMGs) is one of the active topics in research. Most MGs exhibit UTS higher than 1,500 MPa due to their glassy nature, while the UTS of most HEAs are lower than 1,000 MPa (Fig. 5d). For data such as the stress ratios that are not related to materials, their statistics are summarized in Fig. 5e. Most tests were conducted under symmetric tension-compression loading with *R* = −1 (76/272) or tension-tension loading with *R* = 0.1 (151/272). Other stress ratios are usually applied in FCG tests. For data reported in types of string, their statistics are summarized in the table for manual examination. For conditions where the sequence of thermomechanical processing is relevant, consecutive steps are paired and examined to obtain their statistics. For MPEAs as an example (Fig. 5f), the processing usually starts with vacuum induction melting (VIM) or arc melting and ends with heat treatment. Techniques such as rolling and rotary swaging can be applied for MPEAs but usually not for MGs since crystallization can be induced. The recorded data show agreement with the domain knowledge and can be used to elucidate the correlation between them.

Incomplete data reporting in the literature due to the lack of norms is common, which brings difficulties in data mining and reduces the credibility or reusability of data. To assess the quality of reported data, a rating score (0-1) is computed as the weighted summation of non-empty entries for each dataset^43^. For the sake of simplicity, we assume equal weights for all the entries. We find that most datasets are rated with scores ranging from 0.6 to 0.95 since not all of the data entries are documented. The rating scores of MGs and MPEAs are similar to the AM alloys^43^ and lower than authorized databases for conventional alloys. Standards for reporting data in journals, conference proceedings, and technical reports should be developed to further improve the quality of databases constructed from literature.

## Usage Notes

**Fig. 6:**
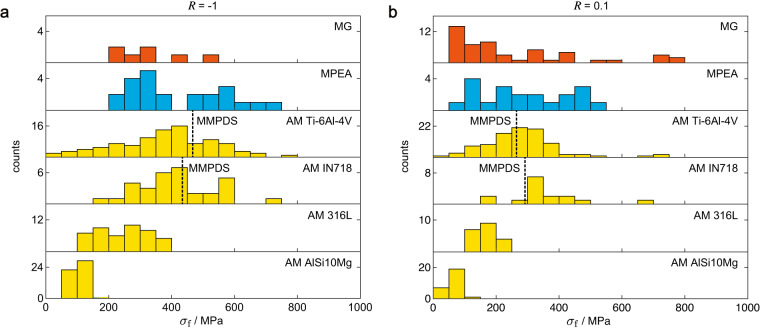
Fatigue strengths derived from the *S*-*N* data. Fatigue strength (FS) at 10^6^ cycles, *σ*_f_, is calculated from *S*-*N* datasets for metallic glasses (MGs), multi-principal element alloys (MPEAs), and additively manufactured (AMed) alloys under stress ratio *R* = −1 (**a**) and 0.1 (**b**). For Ti-6Al-4V and IN718, MMPDS data are added as ref. ^86^.

In experiments, material properties such as FS^85^, fatigue limit (FL)^86^, and glass transition temperature^87^ are derived from the raw data and affected by the post-processing techniques of data filtering and fitting. It is thus important to record the raw data in the database as well as the derived values. In industry, the FS at *N* loading cycles and fatigue limit (FL) are of interest and are usually derived from the discrete *S*-*N* data. However, FL is not well-defined in practice^88,89^, which depends on the run-out cycle chosen in the tests by considering the experimental duration and budget. The staircase method^90^ is commonly used to determine the FL but requires a lot of samples and tests, which is expensive and rarely applied for fatigue tests of new materials. The reported FL in the literature is usually calculated from *S*-*N* curves at the run-out cycle or the value of the run-out data. The values of FL thus vary by their definitions and the fitting procedures for specific *S*-*N* datasets. The problem applies to FS as well. A direct comparison of FL or FS reported in the articles could thus be inaccurate. To address this issue, we provide a MATLAB script (cal_property.m) to calculate their values from the *S*-*N* data. Linear and Stromeryer models^91^ are developed, and the run-out data can be optionally included or not^43^. Using the linear model without run-out data as an example, the FSs of MGs, MPEAs, and AM alloys at 10^6^ cycles (*R* = −1 and 0.1) are summarized in Fig. 6. The results show that the FSs of MGs and MPEAs are higher than that of AM 316 L and AlSi10Mg, and comparable with those of AM Ti-6Al-4V and IN718. MGs and MPEAs still have great potential considering their much richer space of material design. In addition, the YS and UTS of MGs are usually much higher than the conventional and AM alloys. MPEAs show comparable UTS to that of conventional and AM alloys but improved ductility^17,92^. Values of mechanical properties such as strength (YS, UTS) and elongation at fracture are recorded in the current database if they are available in the fatigue-related literature we collected, which can be integrated with records published in other databases^38,93^ for a more complete picture of the strength-ductility relationship.

Access by APIs is essential for the design of reusable databases with high fidelity. For example, users can use the script (cal_property.m) to extract records of material properties that depend on the post-processing techniques, such as FS. A template script (add_entry.m) is provided to add data entries such as densities, toughness, and hardness. Data published after the release of our database can be formatted into the unified language of fatigue data (ULFD)^43^ and directly imported using the script (import_ulfd.m). For dataset scoring, we provide a script (cal_rate_score.m) for the users to define the weight of each data entry.

The FatigueData-CMA2022 database and associated APIs lay the foundation for the exploration of fatigue-resistant MGs and MPEAs in their rather complex space of design. The automated workflow is improved from our previous work^43^, specifically on figure segmentation, figure classification, and text data extraction. Our results also demonstrate the capability of LLMs such as GPT on the construction of databases in material sciences, and can guide future studies on the fine-tuning of LLMs or developing alternative machine-learning techniques to understand the CPMP relationship of complex metallic alloys.

## Data Availability

The scripts used to extract information from figures, tables, and text are mainly based on open-source codes and models including ResNet^64^, table extractor^65^, and Simple Transformers. The in-house scripts for data extraction and analysis are publicly released at the GitHub repository (https://github.com/xuzpgroup/ZianZhang/tree/main/FatigueData-CMA2022 and https://github.com/xuzpgroup/ZianZhang/tree/main/FatigueData-AM2022), which can be used by acknowledging the current article and under the MIT license. These scripts include a detailed, step-by-step tutorial for the use of the database published with this article.
